# Sickness absence and disability pension after road traffic accidents, a nationwide register-based study comparing different road user groups with matched references

**DOI:** 10.1016/j.heliyon.2024.e28596

**Published:** 2024-03-23

**Authors:** Linnea Kjeldgård, Helena Stigson, Kristin Farrants, Emilie Friberg

**Affiliations:** a Division of Insurance Medicine, Department of Clinical Neuroscience, Karolinska Institutet, SE-171 77, Stockholm, Sweden; b Division of Vehicle Safety, Mechanics and Maritime Sciences, Chalmers University of Technology, Gothenburg, Sweden; c Folksam Research, Folksam Insurance Group, Stockholm, Sweden

**Keywords:** Sick leave, Traffic injury, Fall accidents, Car occupants, Bicyclists, Population-based

## Abstract

**Background:**

Being injured in a road traffic accident may affect individuals’ functional ability and in turn lead to sickness absence (SA) and disability pension (DP). Knowledge regarding long-term consequences in terms of SA and DP following a road traffic accident is lacking, especially comparing different groups of road users and compared to the general population. The aim was to estimate excess diagnosis-specific SA and DP among individuals of different road user groups injured in a road traffic accident compared to matched references without such injury.

**Methods:**

A nationwide register-based study, including all working individuals aged 20–59 years and living in Sweden who in 2015 had in- or specialized outpatient healthcare after a new traffic-related injury (n = 20 177) and population-based matched references (matched on: sex, age, level of education, country of birth, living in cities) without any traffic-related injury during 2014–2015 (n = 100 885). Diagnosis-specific (injury and other diagnoses) SA and DP were assessed during 5 years: 1 year before and 4 years following the accident. Mean SA and DP net days/year for each road user group and mean differences of (excess) SA and DP net days/year compared with their matched references were calculated with independent t-tests with bootstrapped 95% confidence intervals (CIs).

**Results:**

A third of all injured road users were bicyclists, 31% were car occupants, 16% were pedestrians (including fall accidents), and 19% were other and unspecified accidents. Pedestrians and other road users were the groups with the highest mean number of SA days during the first year following the accident (51 and 49 days/year respectively). The matched references had between 8 and 13 SA days/year throughout the study period. The excess SA days/year were elevated for all road user groups all five studied years. Excess SA due to injury diagnoses was 15–35 days/year during the first year following the accident. Excess SA due to diagnoses other than injuries were about eight days/year during the whole study period for pedestrians and car occupants and about zero for the bicyclists. The excess DP was low, although it increased every year after the accident for pedestrians and car occupants; for bicyclists no excess DP was seen.

**Conclusion:**

Higher levels of SA due to injury diagnoses were seen among all road user groups during the first year after the accident compared to their references. Pedestrians and car occupants had more excess SA due to other diagnoses and more excess DP four years after the accident than bicyclists and other road users.

## 1 Introduction

Globally, road traffic injuries contribute to 1.3 million deaths per year [1]. In addition, between 20 and 50 million individuals suffer non-fatal injuries which could lead to disability as a result of their injury [1]. Road traffic injuries were the sixth leading cause of disability-adjusted life years (DALYs) in 2019 [1]. The UN’s global goals on sustainability (SDG) strives both for safer and more sustainable transportations [1,2]. Likewise, in Sweden, the Vision Zero has the long-term vision that no one should die or suffer injuries leading to long-term consequences within the road transport system [3], it was adopted in 1997 and has since then been recognized in several countries and has inspired road safety initiatives in many parts of the world.

Sustaining an injury in a road traffic accident may affect the individuals’ work ability and lead to sickness absence (SA) and/or disability pension (DP). It has previously been shown that about a fifth of individuals in a road traffic accident (pedestrians, bicyclists, and car occupants) had a new SA spell in connection to an accident [[4], [5], [6]]. In addition, the long-term consequences in terms of SA and DP for pedestrians, bicyclists and car occupants have been studied, showing heterogeneity in duration, reoccurrence, and diagnosis of SA and DP during the years following the accident [[7], [8], [9]]. However, SA and DP after a road traffic accident have not been studied in relation to the general population.

The aim was to estimate excess diagnosis-specific SA and DP net days/year among individuals injured in a road traffic accident compared to matched references without such injury, for different road user groups, during five years: one year before and four years after the accident, in order to map the long-term consequences in terms of SA and DP among individuals injured in traffic-related accidents compared to individuals without such injuries.

## 2 Materials and methods

A prospective cohort study was conducted, including all working individuals, 20–59 years of age in Sweden injured in a road traffic accident in 2015. Annual net days of SA and DP were analyzed during five years, one year before (Y_-1_) and four years after (Y_+4_) the date of the injury (T_0_) and was compared to non-injured matched references.

All individuals living in Sweden, ≥16 years old, and with income from work, unemployment, or parental-leave benefits can apply for SA benefits from the Social Insurance Agency if they have a disease or injury that leads to reduced work capacity [10]. The first day of a SA spell is an unreimbursed qualifying day (more days for self-employed). A physician’s certificate is required after day 7. For employees, day 2–14 are reimbursed by the employer [10]. For others, e.g., unemployed, the Social Insurance Agency administrates the benefits from the second day of SA, thus information on shorter SA spells was available for these individuals. In order not to introduce a bias, only information on SA spells >14 days was used. All individuals aged 19–64 can be granted DP if disease or injury leads to long-term or permanent work incapacity. Both SA and DP can be granted for full- or part-time (100, 75, 50, 25%) of ordinary work hours. That means that someone on part-time DP can have part-time SA at the same time.

Microdata from several nationwide registers were used and linked at the individual level, using the unique personal identity number assigned to all residents in Sweden [9].

- The in- and specialized outpatient registers, from the *National Board of Health and Welfare*, were used to identify those injured in a road traffic accident and for medical information related to the injury as well as for comorbidity.
- The Cause of Death Register, from the *National Board of Health and Welfare*, was used to identify those who died during the study period.
- The Longitudinal Integration Database for Health Insurance and Labour Market Studies (LISA), from *Statistics Sweden*, was used to identify the source population all individuals living in Sweden 31 December 2014 and information on sociodemographic factors (sex, age, educational level, country of birth, type of living area, marital status, in paid work) also measured 31 December 2014.
- Micro-data for Analyses of the Social Insurance (MiDAS), from the *Swedish Social Insurance Agency*, was used for information on dates and diagnoses of SA and DP.

### 2.1 Study population

**Fig. 1:**
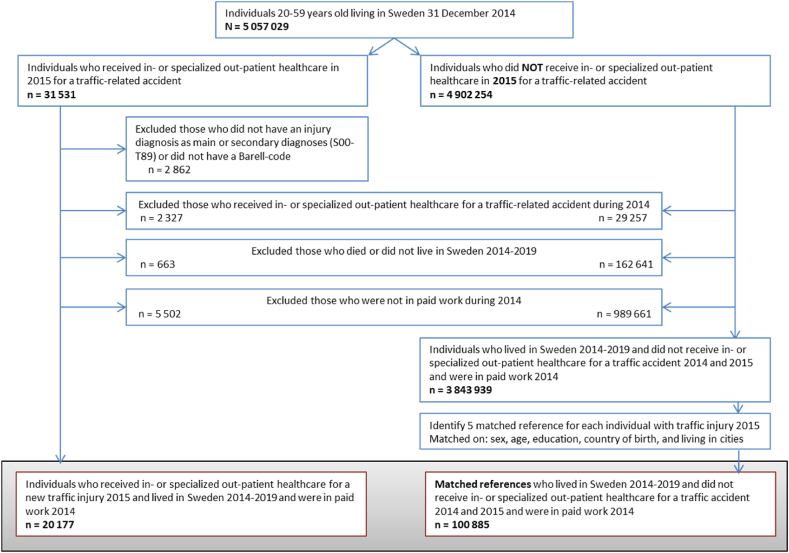
Flowchart of study population, inclusion and exclusion criteria.

All individuals 20–59 years old and living in Sweden 31 December 2014 who during 2015 had at least one hospitalization or visit in specialized outpatient healthcare due to a road traffic accident (International Statistical Classification of Diseases and Related Health Problems; ICD-10 [8]: V01–V79, V80.2-V80.5, V82, V83.0-V83.3,V84.0-V84.3, V85.0-V85.3, V86.0-V86.3, V87, V89.2, V89.3, V89.9, W00.4, W01.4, W02.4, W03.4, W04.4, W05.4, W10.4, W15.4, W17.4, W18.4, W19.4, W51.4) were included. Individuals who did not have an injury diagnosis (ICD-10: S00-T88) or couldn’t be classified according to the Barell-classification [11] were excluded. Individuals who had any traffic related in- or outpatient healthcare (ICD-10: V01–V79, V80.2-V80.5, V82, V83.0-V83.3,V84.0-V84.3, V85.0-V85.3, V86.0-V86.3, V87, V89.2, V89.3, V89.9, W00.4, W01.4, W02.4, W03.4, W04.4, W05.4, W10.4, W15.4, W17.4, W18.4, W19.4, W51.4) during 2014 and those not living in Sweden during 2014–2019 were excluded. In addition, those not in paid work according to labour market status in LISA during 2014 were excluded. To enable comparisons between groups that may differ in baseline characteristics, each individual was matched (exact matching without replacement) with five references without any road traffic accident during 2014 and 2015, living in Sweden 2014–2019, and were in paid work 2014 (Fig. 1). Individuals were matched on: sex (women; men), year of birth (year), level of education (elementary/high school (≤12 years); university/college (>12 years)), country of birth (Sweden; not Sweden), and living in cities (yes; no).

The date of the accident, denoted as T_0,_ refers to the first date of the in- or specialized outpatient healthcare visit/hospitalization, as the actual date of their accident/fall is not included in the registers. For the matched references, T_0_ refers to the date of T_0_ for the injured individual.

Number of SA and DP net days were assessed yearly during a period of five years; one year before and four years after the accident date, i.e., T_0_. Diagnoses of SA and DP were categorized as: Injuries (S00-T98); and Other diagnoses (all SA except S00-T98). Furthermore, in some analyses the SA and DP diagnoses were categorized into seven groups: Injuries (S00-T98), Cancer (C00-D48), Mental diseases (F00–F99), Central Nervous System (CNS) (G00-G99), Cardiovascular disease (CVD) (I00–I99), Musculoskeletal (M00-M99), Other (all other SA).

Net days of SA and DP were used, i.e., partial days of SA were combined, e.g., two days of part-time SA for 50% were summed to one net day, and similarly, partial days of DP were combined to net days of DP.

The accidents were categorized into four road user groups: pedestrians (including fall accidents) (V01–V09, W00.4, W01.4, W02.4, W03.4, W04.4, W05.4, W10.4, W15.4, W17.4, W18.4, W19.4, W51.4); bicyclists (V10–V19); car occupants (V40–V49); and other road users (Motor cyclists, mopeds, truck drivers, bus occupants, 3-wheelers, equestrians, trams, other vehicles) (V20–V39, V50–V79, V80.2-V80.5, V82, V83.0-V83.3, V84.0-V84.3, V85.0-V85.3, V86.0-V86.3, V87, V89.2, V89.3, V89.9).

The main diagnosis and the secondary diagnoses were categorized using a modified version of the Barell matrix [11], into categories of injured body region and type of injury. Most of the individuals had only one injury diagnosis, but for those individuals who had several, the main diagnosis was prioritized before any of the secondary diagnoses. Some individuals had up to six visits/hospitalizations at T_0._ In these cases, the injury diagnoses from inpatient healthcare were prioritized over those from outpatient healthcare. In addition, an injury with ICD10: S00–S99 was prioritized over an injury with the ICD10: T00-T88.

The injury was categorized as Injured body region into the following twelve groups: ‘Head, face, and neck, not Traumatic Brain Injury (TBI)’; ‘TBI, not concussion’; ‘Concussion’; ‘Vertebral column and spinal cord’; ‘Torso’; ‘Shoulder and upper arm’; ‘Forearm and elbow’; ‘Wrist, hand, and other arm’; ‘Hip, upper leg, and thigh’; ‘Knee’; ‘Lower leg, ankle, foot, and other leg’; and ‘Other and unspecified’. Moreover, the injury was also categorized as Type of injury into six groups: Fracture; Dislocation; Sprains and strains; Internal (brain, spinal cord, and other internal organs); External (open wounds, contusions, and superficial injuries); and Other and unspecified. Similar categorizations were used in recent studies on injuries among different road user groups [[4], [5], [6], [7],^12^].

Healthcare at the inclusion date (T_0_) was categorized into: only specialized outpatient healthcare; inpatient ≤2 days; and inpatient healthcare >2 days. If someone had both specialized outpatient healthcare and inpatient healthcare at T_0_ they were categorized as inpatient healthcare.

Age was categorized as: 20–39; 40–59 years, and marital status as: married; not married. Comorbidity was measured during Y_-1_ as having any hospitalization or specialized outpatient healthcare due to: mental diagnoses (F00–F99); musculoskeletal diagnoses (M00-M99) or other diagnoses (other than: F00–F99, M00-M99, O80, S00-T98, and Z00-Z99).

### 2.2 Statistical analyses

Descriptive statistics of the study population were calculated stratified by road user group and injured/matched references. Mean SA and DP net days/year for each road user group and mean differences of SA and DP (i.e. excess) net days/year compared with their matched references were calculated using independent t-tests with bootstrapped 95% confidence intervals (CIs).

The statistical analyses were performed using SAS (version 9.4) and R (version 4.2.1).

## 3 Results

There were 20 177 working individuals aged 20–59 years with in- or specialized outpatient healthcare due to a new traffic accident including fall accidents in 2015, with 100 885 matched references. A third of the injured road users were bicyclists, 31% car occupants, 16% pedestrians (including fall accidents), and 19% were other road users (of which most were motorcycle/moped riders 82%).

There were higher proportions of individuals in the older age groups among pedestrians and bicyclists than among car occupants and other road users. A higher proportion of the pedestrians were women, while there was a higher proportion of men among the bicyclists, car occupants and other road users, especially among other road users, where 85% were men (Table 1). Among other road users there was a higher proportion who had inpatient healthcare.

**Table 1:** Characteristics (number and percentages) of the study population aged 20–59 with a road traffic injury in 2015 and their matched references, by road user group.

	Pedestrians		Bicyclists		Car occupants		Other road user groups		Overall		Overall, total
	Injured	References	Injured	References	Injured	References	Injured	References	Injured	References	
	n (%)	n (%)	n (%)	n (%)	n (%)	n (%)	n (%)	n (%)	n (%)	n (%)	n (%)
**Total**	**3211**	**16 055**	**6997**	**34 985**	**6221**	**31 105**	**3748**	**18 740**	**20 177**	**100 885**	**121 062**
**Sex**^a^											
Men	1382 (43.0)	6910 (43.0)	4186 (59.8)	20 930 (59.8)	3262 (52.4)	16 310 (52.4)	3188 (85.1)	15 940 (85.1)	12 018 (59.6)	60 090 (59.6)	72 108 (59.6)
Women	1829 (57.0)	9145 (57.0)	2811 (40.2)	14 055 (40.2)	2959 (47.6)	14 795 (47.6)	560 (14.9)	2800 (14.9)	8159 (40.4)	40 795 (40.4)	48 954 (40.4)
**Mean age (SD)**^a^	42.4 (12.1)	42.4 (12.1)	41.4 (11.0)	41.4 (11.0)	35.7 (11.4)	35.7 (11.4)	36.8 (11.4)	36.8 (11.4)	39.0 (11.7)	39.0 (11.7)	39.0 (11.7)
**Age group, years**											
20-39	1227 (38.2)	6135 (38.2)	2881 (41.2)	14 405 (41.2)	3883 (62.4)	19 415 (62.4)	2149 (57.3)	10 745 (57.3)	10 140 (50.3)	50 700 (50.3)	60 840 (50.3)
40-59	1984 (61.8)	9920 (61.8)	4116 (58.8)	20 580 (58.8)	2338 (37.6)	11 690 (37.6)	1599 (42.7)	7995 (42.7)	10 037 (49.7)	50 185 (49.7)	60 222 (49.7)
**Level of education**^a^											
University/college	1157 (36.0)	5785 (36.0)	3354 (47.9)	16 770 (47.9)	1902 (30.6)	9510 (30.6)	741 (19.8)	3705 (19.8)	7154 (35.5)	35 770 (35.5)	42 924 (35.5)
Elementary/High school	2054 (64.0)	10 270 (64.0)	3643 (52.1)	18 215 (52.1)	4319 (69.4)	21 595 (69.4)	3007 (80.2)	15 035 (80.2)	13 023 (64.5)	65 115 (64.5)	78 138 (64.5)
**Living in cities**^a^											
Yes	1257 (39.1)	6285 (39.1)	3222 (46.0)	16 110 (46.0)	2186 (35.1)	10 930 (35.1)	1162 (31.0)	5810 (31.0)	7827 (38.8)	39 135 (38.8)	46 962 (38.8)
No	1954 (60.9)	9770 (60.9)	3775 (54.0)	18 875 (54.0)	4035 (64.9)	20 175 (64.9)	2586 (69.0)	12 930 (69.0)	12 350 (61.2)	61 750 (61.2)	74 100 (61.2)
**Country of birth**^a^											
Sweden	2698 (84.0)	13 490 (84.0)	6076 (86.8)	30 380 (86.8)	4916 (79.0)	24 580 (79.0)	3359 (89.6)	16 795 (89.6)	17 049 (84.5)	85 245 (84.5)	102 294 (84.5)
Not Sweden	513 (16.0)	2565 (16.0)	921 (13.2)	4605 (13.2)	1305 (21.0)	6525 (21.0)	389 (10.4)	1945 (10.4)	3128 (15.5)	15 640 (15.5)	18 768 (15.5)
**Married**											
No	1993 (62.1)	9212 (57.4)	4175 (59.7)	20 082 (57.4)	4172 (67.1)	20 462 (65.8)	2740 (73.1)	12 718 (67.9)	13 080 (64.8)	62 474 (61.9)	75 554 (62.4)
Yes	1218 (37.9)	6843 (42.6)	2822 (40.3)	14 903 (42.6)	2049 (32.9)	10 643 (34.2)	1008 (26.9)	6022 (32.1)	7097 (35.2)	38 411 (38.1)	45 508 (37.6)
**Mental comorbidity**											
No	3019 (94.0)	15 561 (96.9)	6700 (95.8)	34 023 (97.3)	5828 (93.7)	30 136 (96.9)	3558 (94.9)	18 221 (97.2)	19 105 (94.7)	97 941 (97.1)	117 046 (96.7)
Yes	192 (6.0)	494 (3.1)	297 (4.2)	962 (2.7)	393 (6.3)	969 (3.1)	190 (5.1)	519 (2.8)	1072 (5.3)	2944 (2.9)	4016 (3.3)
**Musculoskeletal comorbidity**											
No	2847 (88.7)	14 921 (92.9)	6435 (92.0)	32 885 (94.0)	5656 (90.9)	29 387 (94.5)	3399 (90.7)	17 773 (94.8)	18 337 (90.9)	94 966 (94.1)	113 303 (93.6)
Yes	364 (11.3)	1134 (7.1)	562 (8.0)	2100 (6.0)	565 (9.1)	1718 (5.5)	349 (9.3)	967 (5.2)	1840 (9.1)	5919 (5.9)	7759 (6.4)
**Others comorbidities**											
No	2203 (68.6)	11 639 (72.5)	5053 (72.2)	26 167 (74.8)	4100 (65.9)	23 142 (74.4)	2845 (75.9)	14 954 (79.8)	14 201 (70.4)	75 902 (75.2)	90 103 (74.4)
Yes	1008 (31.4)	4416 (27.5)	1944 (27.8)	8818 (25.2)	2121 (34.1)	7963 (25.6)	903 (24.1)	3786 (20.2)	5976 (29.6)	24 983 (24.8)	30 959 (25.6)
**Healthcare**											
Only specialized outpatient	2809 (87.5)		6137 (87.7)		5454 (87.7)		3017 (80.5)		17 417 (86.3)		
Inpatient ≤2	257 (8.0)		601 (8.6)		623 (10.0)		471 (12.6)		1952 (9.7)		
Inpatient >2	145 (4.5)		259 (3.7)		144 (2.3)		260 (6.9)		808 (4.0)		

a Matched variable.

Comorbidity the year before the accident was more common among those injured in a road traffic accident compared to their references. Six percent of pedestrians and car occupants had in- or specialized outpatient healthcare due to mental diagnoses the year before the accident, twice that of their matched references (3%) (Table 1). On average, nine percent among those injured in a road traffic accident had healthcare due to musculoskeletal diagnoses the year before the accident, while the corresponding number for the references was six percent.

In total, the most common injured body regions were wrist and hand, and injuries to the ‘head, face, and neck, not TBI’. The most common injury type were external injuries and fractures (Table A1). However, the injured body region and type of injuries varied among the road user groups. The most common injuries for pedestrians and bicyclists were external injuries to the ‘head, face, and neck, not TBI’ and fractures to the upper extremities. For pedestrians, fractures to the ‘wrist, hand, and other arm’ and fractures to the ‘lower leg, ankle, foot, and other leg’ were the most common. For car occupants the most common injuries were sprains and strains in the ‘vertebral column and spinal cord’ (including whiplash), and external injuries to the ‘head, face, and neck, not TBI’ and to the ‘torso’. Similarly to the pedestrians and bicyclists, among other road users, fractures to the ‘shoulder and upper arm’, to the ‘wrist, hand, and other arm’ and, to the ‘lower leg, ankle, foot, and other leg’ were most common, while there was a lower proportion of injuries to the head, face, and neck.

**Fig. 2:**
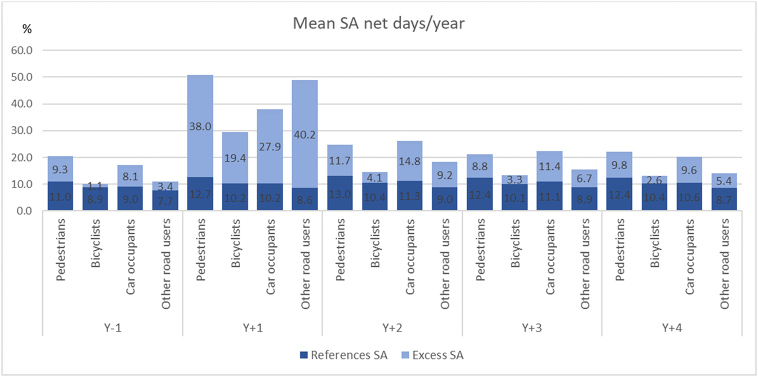
Average net days/year of sickness absence (SA) for the references and excess SA for the injured in the different road user groups.

Pedestrians and other road users were the road user groups with the highest excess SA net days during the first year following the accident (38 days/year, and 40 days/year respectively) (Fig. 2). The second year after the accident pedestrians and car occupants had the highest excess SA net days (12 and 15 days/year, respectively), as well as in the third and fourth year after the accident. In addition, these two road user groups had most excess SA the year before the accident (9 and 8 days/year, respectively). The matched references had 8-13 net days of SA per year, during the whole study period (Fig. 2).

**Fig. 3:**
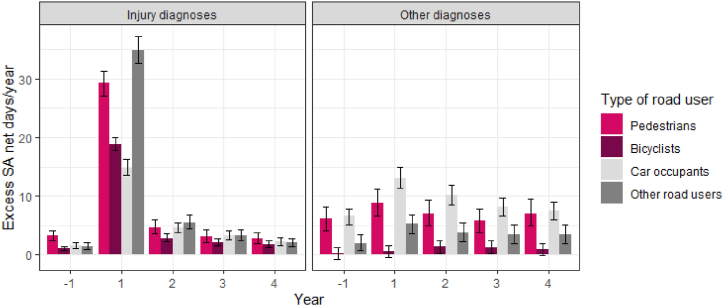
Excess sickness absence (SA) net days/year due to injury diagnoses (left) and other diagnoses (right) before and after the accident in the different road user groups compared to their matched references and 95% bootstrapped confidence intervals.

**Fig. 4:**
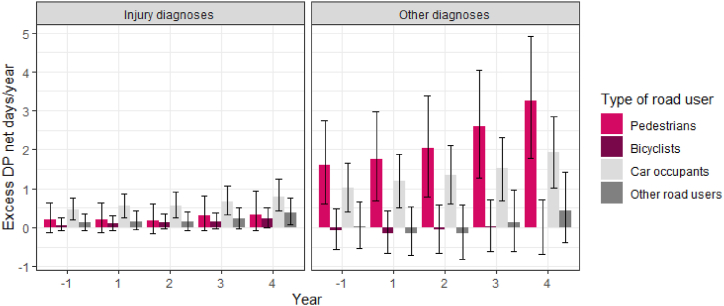
Excess disability pension (DP) net days/year due to injury diagnoses (left) and other diagnoses (right) before and after the accident in the different road user groups compared to their matched references and 95% bootstrapped confidence intervals.

Examining excess SA by injury and other diagnosis of SA and DP shows that excess SA due to injury diagnoses was especially high during the first year following the accident for all road user groups, and was still elevated during the following years compared to their references (Fig. 3). Pedestrians and car occupants had more excess SA due to other diagnoses than bicyclists and other road users each year. They also had higher and increasing excess DP due to other diagnoses, whereas bicyclists and other road users had no excess DP due to other diagnoses (Fig. 4). Car occupants was the road user group with the lowest excess SA due to injury diagnoses in the first year, but they had the highest amount of excess SA due to other diagnoses that year.

**Fig. 5:**
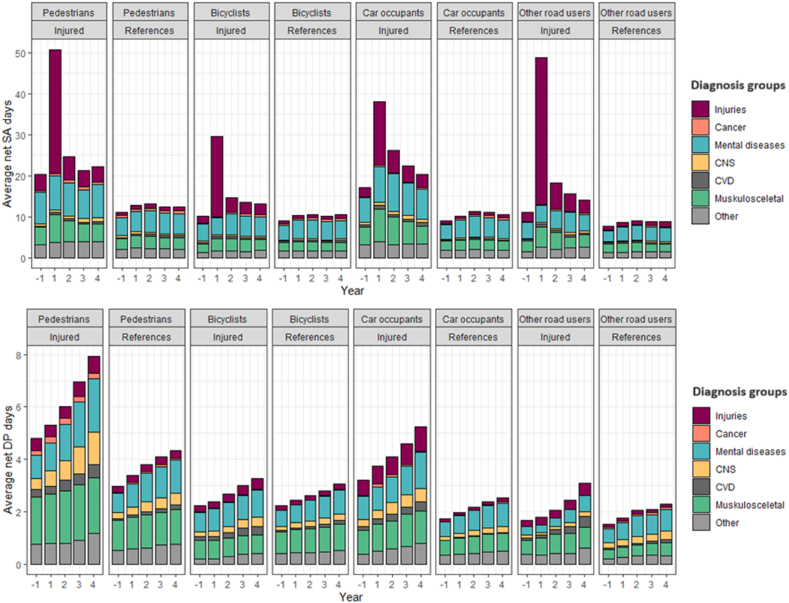
Average SA (top panels) and DP (bottom panels) net days/year due to different diagnoses before and after the accident for the different road user groups and their matched references. Abbreviations: SA: Sickness absence; DP: Disability pension; CNS: Central nervous system; CVD: Cardiovascular diseases.

The highest average net SA and DP days/year were due to mental diagnoses and musculoskeletal diagnoses, except for among the injured individuals, where SA due to injury diagnoses accounted for the highest number of net days during the first year after the accident (Fig. 5).

## 4 Discussion

In this nationwide register study, injured individuals in all road user groups had high excess SA compared to their references during the year after a road traffic accident. This was highest among the group other road users and lowest among bicyclists. All the following years, pedestrians and car occupants had the highest excess SA and DP (especially due to diagnoses other than injuries) compared to their references.

The sociodemographic characteristics in the road user groups varied. Injured pedestrians were older and a higher proportion of them were women, while injured bicyclists were older and a higher proportion were men. Injured car occupants were younger, and other injured road users were younger and consisted primarily of men. These differences between the road user groups could depend on several factors regarding which individuals engage in walking, bicycling, or driving, to what extent they use the transportation type, how likely they are to get involved in an accident, and how likely they are to become injured and seek healthcare if they are injured. For example, among pedestrians, especially in relation to fall accidents, older individuals are generally overrepresented [13] and more likely to get a fracture when falling [14]. For this reason, each road user group were compared to a group of matched references, rather than compared directly to each other. However, it is possible that there are still other compositional factors between the road user groups that might be related to their risk of SA or DP that were not accounted for in the matching.

In addition, the types of injuries varied between the road user groups. As previously shown, different types of injuries lead to different amounts and/or durations of SA [4,6,9,12]. For example, injuries to the upper extremities, the vertebral column or spinal cord were associated with long-term SA among injured pedestrians [9]. For injured bicyclist injuries to the lower leg, shoulder, upper arm and TBI were associated with longer duration of SA [7,12]. Among injured car occupants, TBI, injuries to the to the upper extremities, vertebral column and spinal cord have been showed to be associated with new SA after a road traffic accident [6]. Different injuries have different implications for SA and DP both due to the time it takes to heal from the injury (for example, if it involves a joint, the rehabilitation tends to be longer) and due to their different impact on work ability (for example, depending on the type of occupation, injuries to various body parts might have more or less impact on an individual’s ability to carry out the work tasks). More pedestrians had fractures and injuries to the wrist and ankle and, as shown by previous findings, most of the pedestrian injuries are from falls [4,9] (e.g., tripping in slippery shoes or slipping and catching themselves with their hands). More bicyclists had injuries to the upper extremities, while head injuries and injuries to the ‘vertebral column and spinal cord’, and ‘torso’ were more common among the car occupants. This is also in line with previous findings [[5], [6], [7], [8],^15^]. Other road users (including mostly motorcyclists) had a lower proportion of injuries to the head, face, and neck in the present study. Injuries to the ‘head, face, and neck, not TBI’ are often external facial injuries, and have previously been shown to have low proportions of SA [5,7]. The different proportions of injuries and their location could be explained by, e.g., type of accident and crash severity. Another aspect that impacts the need for SA and DP is not just the type and location of the injury, but also the severity. Other road users had a higher proportion of inpatient healthcare, which might indicate more severe injuries. The lower proportion of injuries to the ‘head, face, and neck, not TBI’ among other road users could be due to the higher proportion of helmet use among motorcyclists compared to bicyclists, but it could also be due to higher crash severity and hence more severe injuries [16] that require longer time to heal and have more impact on work ability.

The differences between road user groups are important to consider when interpreting the results., i.e., the compositions of the road user groups, the risk of sustaining a certain injury, and the consequences of the injury (e.g., need of healthcare or SA) varies. For example, higher age and female gender are risk factors for SA and DP both in general [17] and after a road traffic injury [[4], [5], [6], [7],^9^,[18], [19], [20], [21]]. A study from Australia on work absence following a road traffic crash found lower risks for prolonged work absence among bicyclists and motorcyclists compared to car occupants [21]. A study from France on 581 individuals injured in a road traffic accident did not find any significant differences between road user groups, even though a higher proportion among those with a late return to work were motorcyclists and a higher proportion among those with an earlier return to work were bicyclists [22]. In order to generalize the results between countries, aspects of the welfare systems, road environment, and other macro-level factors (e.g., GDP, the labour market, or accessibility legislation) that impact both the occurrence and consequences of accidents have to be considered; hence one might argue that the study from France may be more directly comparable to our findings than the one from Australia, as the Australian welfare system differs more from the Swedish. On the other hand, the injuries that have the most impact on the individuals are probably the same types of injuries, regardless of which country the individuals are living in, even if the social security may vary between countries.

The present study found that not only pedestrians but also all other road user groups had higher comorbidity the year before the accident than their matched references. The date of the in- or specialized outpatient healthcare was used as the date of the accident, as the actual date of the accident is not recorded in the register. That is, the accident may in some cases have occurred some days before the registered date of in- and specialized outpatient healthcare if the individual waited to seek healthcare, or if he or she sought primary healthcare first, and was only later referred to specialist healthcare. This would lead to an overestimation of SA the year before the accident, and potentially also an overestimation of the differences between the road user groups, if some road user groups were more likely to delay seeking healthcare than others. To handle this, the last month of comorbidity before the accident was excluded in a sensitivity analysis, which did not alter the results substantially. Further, the injured individuals had excess SA and DP due to other diagnoses before the accident, especially pedestrians and car occupants. Hence, those who were injured had a higher burden of disease and comorbidity before the accident than those not involved in a road accident. This finding is in line with previous findings that observed that previous disability is a risk factor for pedestrians being involved in and injured in a fall or in a collision with another road user [23]. Prior morbidity may prolong rehabilitation after an injury, and could be one of the reasons why especially pedestrians and car occupants had excess SA and DP throughout the entire follow-up. Alternatively, their excess SA and DP during the follow-up could be due to the prior comorbidity, rather than the injury itself. It is worth noting that bicyclists were the road user group with the lowest proportion of comorbidity, and they also had the lowest excess SA the year before the accident as well as very little excess SA and even negative excess DP during the follow-up. This could imply better health and faster recovery among the bicyclists than among the other road user groups. This is in line with previous findings that the benefits from bicycling outweigh the negatives from being involved in a crash. Bicyclists are generally in better health (physically, mentally and quality of life) than those who do not cycle and thus might have a better starting point to handle the potential health issue that a crash could lead to [24].

The results from this study highlight the importance of improving the safety for all road user groups to prevent excess SA and DP. Our results also indicate that particular attention should be paid to pedestrians and car occupants, as they had higher excess SA and DP during the follow-up than other road user groups. Reducing excess SA and DP from road traffic injuries requires a two-pronged approach: both preventing accidents and reducing the negative outcomes of the accidents. It is also important to improve accessibility and safety for individuals with different kinds of morbidity and functional limitations, as those who were injured in an accident had higher levels of prior morbidity than their matched references. Prior morbidity is also a risk factor for subsequent SA and DP, and can thus have exacerbated the consequences of the accident in terms of SA and DP. Personal devices such as reflectors, good shoes, or higher car safety levels [25] could reduce both the risk of accidents and the risk of injury. Furthermore, measures related to the traffic environment such as gritting/salting slippery roads, improved pavement or road materials, lowering curbs, or improving the separation between road user groups can improve the safety and thereby reduce both the risk for being involved in an accident and being injured from any accidents. Work adaptations can reduce the need for SA and DP among injured individuals, although they are not always possible. Sickness absence and DP are granted if the work ability is reduced, while improving the road traffic safety may reduce the need for SA and DP and hence more individuals can contribute to society.

One of the main strengths of this study is the use of high-quality nationwide register data, with total population coverage, several years of follow-up, and that the results were not hampered by recall bias [26]. Another strength is that all injuries from road traffic accidents severe enough to require in- or outpatient healthcare were included and that the outcome of SA and DP could be studied in relation to the general population without a road traffic injury (i.e., matched references with same proportion of age, sex, level of education, born in Sweden and living in cities as within each road user group). The large number of included individuals allowed for more detailed analyses regarding different factors and differences in excess SA and DP and the diagnoses of such SA and DP. More detailed analyses of the injured individuals’ previous comorbidity in relation to their SA and DP compared to individuals without road traffic injuries would be beneficial for future research. It would also be beneficial to include primary healthcare to capture the impact of minor injuries.

## 5 Conclusions

This nationwide register study of individuals injured in a road traffic accident and their population-based matched references observed higher levels of SA due to injury diagnoses among all road user groups the first year after the accident compared to their references. Pedestrians and car occupants had more excess SA due to other diagnoses and more excess DP throughout the study period, while bicyclists had no such excess SA or DP compared to their references. These results highlight the importance of further investigating the differences in outcomes between injured road user groups and what factors may affect these outcomes.

## Ethics approval and consent to participate

The project was approved by the Regional Ethical Review Board in Stockholm, Sweden (Dnr 2021–03628, 2007/762-31, 2009/23–32, 2009/1917-32, 2011/806-32, 2011/1710-32, 2016/1533-32). All methods were carried out in accordance with relevant guidelines and regulations. Consent to participate was not applicable for register data.

## Consent for publication

Not applicable.

## Funding

The study was financially supported by AFA insurance, and we utilised data from the REWHARD consortium supported by the 10.13039/501100004359Swedish Research Council (VR; grant number 2017-00624).

## Data availability statement

The sensitive microdata used in this study is administrated by the Division of Insurance Medicine, Karolinska Institutet, and cannot be shared publicly, according to privacy regulations. According to the General Data Protection Regulation, the Swedish law SFS 2018:218, the Swedish Data Protection Act, the Swedish Ethical Review Act, and the Public Access to Information and Secrecy Act, data can only be made available, after legal review, for researchers who meet the criteria for access to this type of sensitive and confidential data. Readers may contact professor Kristina Alexanderson (kristina.alexanderson@ki.se) regarding the data.

## CRediT authorship contribution statement

**Linnea Kjeldgård:** Writing – original draft, Visualization, Methodology, Investigation, Formal analysis, Data curation, Conceptualization. **Helena Stigson:** Writing – original draft, Supervision, Methodology, Conceptualization. **Kristin Farrants:** Writing – original draft, Supervision, Methodology, Conceptualization. **Emilie Friberg:** Writing – original draft, Supervision, Software, Resources, Project administration, Methodology, Funding acquisition, Conceptualization.

## Declaration of competing interest

The authors declare the following financial interests/personal relationships which may be considered as potential competing interests Emilie Friberg reports financial support was provided by AFA Insurance. If there are other authors, they declare that they have no known competing financial interests or personal relationships that could have appeared to influence the work reported in this paper.

## List of abbreviations

DP: Disability pension
SA: Sickness absence
OR: Odds ratio
CI: Confidence interval
SDG: Sustainable Development Goals
CNS: Central Nervous System
CVD: Cardiovascular Diseases
TBI: Traumatic Brain Injury
