# Supplementary material for: Sickness absence and disability pension after road traffic accidents, a nationwide register-based study comparing different road user groups with matched references
Source: Heliyon. 2024 Mar 23;10(7):e28596. doi: 10.1016/j.heliyon.2024.e28596 (PMC10988042; doi:10.1016/j.heliyon.2024.e28596)
Supplement: Multimedia component 1 [file mmc1.docx]

Table A1. Proportions (%) of injured body region by type of injury in each road user group.

|  |  | Fractures | Dislocation | Sprains and strains | Internal | External | Other and unspecified | Total |
| --- | --- | --- | --- | --- | --- | --- | --- | --- |
| TBI^1^, not concussion | Pedestrians | - | - | - | 1,3 | - | - | 1,3 |
|  | Bicyclists | - | - | - | 1,1 | - | - | 1,1 |
|  | Car occupants | - | - | - | 0,8 | - | - | 0,8 |
|  | Other road users | - | - | - | 1,0 | - | - | 1,0 |
| Concussion | Pedestrians | - | - | - | 6,2 | - | - | 6,2 |
|  | Bicyclists | - | - | - | 6,3 | - | - | 6,3 |
|  | Car occupants | - | - | - | 4,0 | - | - | 4,0 |
|  | Other road users | - | - | - | 3,7 | - | - | 3,7 |
| Head, face, and neck, not TBI^1^ | Pedestrians | 1,4 | Too few | Too few | - | 10,4 | 0,7 | 12,6 |
|  | Bicyclists | 2,6 | Too few | Too few | - | 14,5 | 1,0 | 18,3 |
|  | Car occupants | 0,6 | Too few | 0,6 | - | 24,9 | 1,8 | 28,0 |
|  | Other road users | 1,0 | - | Too few | - | 6,0 | 0,7 | 7,8 |
| Vertebral column and spinal cord | Pedestrians | 1,2 | Too few | 0,8 | Too few | - | - | 2,1 |
|  | Bicyclists | 1,4 | Too few | 1,0 | Too few | - | - | 2,4 |
|  | Car occupants | 2,7 | 0,2 | 30,4 | Too few | - | - | 33,4 |
|  | Other road users | 3,5 | - | 3,7 | - | - | - | 7,2 |
| Torso | Pedestrians | 2,2 | - | Too few | Too few | 3,4 | Too few | 6,0 |
|  | Bicyclists | 3,2 | - | Too few | 0,9 | 4,0 | Too few | 8,2 |
|  | Car occupants | 2,4 | - | 0,1 | 0,9 | 13,0 | 0,3 | 16,7 |
|  | Other road users | 5,7 | - | Too few | 1,8 | 5,9 | 0,3 | 13,8 |
| Shoulder and upper arm | Pedestrians | 4,3 | 1,8 | 0,5 | - | 2,2 | Too few | 8,9 |
|  | Bicyclists | 8,9 | 3,5 | 0,8 | - | 4,3 | Too few | 17,6 |
|  | Car occupants | 0,7 | 0,2 | 0,4 | - | 3,5 | Too few | 4,8 |
|  | Other road users | 9,5 | 2,9 | 1,0 | - | 4,1 | Too few | 17,6 |
| Forearm and elbow | Pedestrians | 4,4 | Too few | Too few | - | 2,1 | - | 6,9 |
|  | Bicyclists | 6,2 | 0,5 | 0,2 | - | 2,8 | - | 9,8 |
|  | Car occupants | 0,6 | Too few | Too few | - | 0,8 | - | 1,4 |
|  | Other road users | 2,2 | 0,2 | Too few | - | 1,7 | Too few | 4,3 |
| Wrist, hand, and other arm | Pedestrians | 13,0 | 0,4 | 2,6 | - | 3,8 | Too few | 19,9 |
|  | Bicyclists | 10,5 | 0,9 | 3,2 | - | 5,0 | Too few | 19,7 |
|  | Car occupants | 1,4 | 0,1 | 0,6 | - | 2,5 | 0,2 | 4,9 |
|  | Other road users | 8,3 | 0,5 | 2,1 | - | 3,8 | 0,5 | 15,2 |
| Hip, upper leg, and thigh | Pedestrians | 1,2 | - | 0,4 | - | 1,2 | - | 2,8 |
|  | Bicyclists | 0,8 | - | 0,1 | - | 1,9 | Too few | 2,9 |
|  | Car occupants | 0,2 | Too few | Too few | - | 0,9 | Too few | 1,2 |
|  | Other road users | 1,0 | Too few | 0,3 | - | 2,4 | Too few | 3,8 |
| Knee | Pedestrians | 1,8 | 0,3 | 3,4 | - | 3,7 | - | 9,2 |
|  | Bicyclists | 1,1 | Too few | 1,6 | - | 3,8 | - | 6,5 |
|  | Car occupants | 0,2 | Too few | 0,4 | - | 1,4 | - | 2,0 |
|  | Other road users | 2,1 | Too few | 4,1 | - | 4,1 | - | 10,4 |
| Lower leg, ankle, foot, and other leg | Pedestrians | 12,2 | Too few | 7,3 | - | 3,6 | 0,2 | 23,6 |
|  | Bicyclists | 3,3 | Too few | 1,3 | - | 1,9 | Too few | 6,6 |
|  | Car occupants | 0,8 | Too few | 0,4 | - | 1,0 | Too few | 2,2 |
|  | Other road users | 7,7 | Too few | 2,2 | - | 4,3 | 0,3 | 14,6 |
| Other and unspecified | Pedestrians | - | - | - | - | 0,3 | Too few | 0,4 |
|  | Bicyclists | - | - | - | - | 0,4 | 0,1 | 0,5 |
|  | Car occupants | - | - | - | - | 0,4 | 0,3 | 0,6 |
|  | Other road users | - | - | - | - | 0,5 | 0,3 | 0,7 |
| Total | Pedestrians | 41,6 | 3,1 | 15,4 | 7,8 | 30,9 | 1,2 | 100,0 |
|  | Bicyclists | 38,1 | 5,0 | 8,4 | 8,4 | 38,6 | 1,4 | 100,0 |
|  | Car occupants | 9,7 | 0,7 | 32,9 | 5,7 | 48,4 | 2,7 | 100,0 |
|  | Other road users | 41,0 | 3,9 | 13,6 | 6,5 | 32,7 | 2,3 | 100,0 |

^1^ TBI = Traumatic Brain Injury

| Colour-pattern | Not applicable | Non or too few too show | < 2 % | >=2%  <5% | >=5% <10% | >=10% |
| --- | --- | --- | --- | --- | --- | --- |
